# The Effects of *Coriandrum sativum* L. and *Cucurbita pepo* L. against Gastrointestinal Parasites in Swine: An In Vivo Study

**DOI:** 10.3390/microorganisms11051230

**Published:** 2023-05-06

**Authors:** Mihai-Horia Băieş, Vlad-Dan Cotuţiu, Marina Spînu, Attila Mathe, Anamaria Cozma-Petruț, Doina Miere, Sorana D. Bolboacǎ, Vasile Cozma

**Affiliations:** 1 Department of Parasitology and Parasitic Disease, Faculty of Veterinary Medicine, University of Agricultural Sciences and Veterinary Medicine of Cluj-Napoca, 3-5 Mǎnǎştur Street, 400372 Cluj-Napoca, Romania; mihai-horia.baies@usamvcluj.ro (M.-H.B.); vlad.cotutiu@usamvcluj.ro (V.-D.C.); vasile.cozma@usamvcluj.ro (V.C.); 2 Department of Infectious Diseases, Faculty of Veterinary Medicine, University of Agricultural Sciences and Veterinary Medicine of Cluj-Napoca, 3-5 Mǎnǎştur Street, 400372 Cluj-Napoca, Romania; marina.spinu@gmail.com; 3 Agricultural Research and Development Station of Turda, 27 Agriculturii Street, 401100 Turda, Romania; mate_atta@yahoo.com; 4 Department of Bromatology, Hygiene, Nutrition, Faculty of Pharmacy, “Iuliu Haţieganu” University of Medicine and Pharmacy, 6 Pasteur Street, 400349 Cluj-Napoca, Romania; dmiere@umfcluj.ro; 5 Department of Medical Informatics and Biostatistics, “Iuliu Haţieganu” University of Medicine and Pharmacy, 6 Louis Pasteur Street, 400349 Cluj-Napoca, Romania; sbolboaca@umfcluj.ro; 6 Academy of Agricultural and Forestry Sciences Gheorghe Ionescu-Siseşti (A.S.A.S.), 61 Mǎrǎşti Boulevard, 011464 Bucharest, Romania

**Keywords:** *Coriandrum sativum* L., *Cucurbita pepo* L., gastrointestinal parasites, swine, low-input farms

## Abstract

Parasitic diseases are responsible for substantial losses in reproduction and productivity in swine, creating a major impairment to efficient and profitable livestock management. The use of phytotherapeutic remedies has notably increased over the past decade due to their bioavailability, decreased toxicity, non-polluting nature, and to some extent due to their antiparasitic effect. The aim of this study was to evaluate the antiparasitic potential of *Cucurbita pepo* L. and *Coriandrum sativum* L. against protozoa and nematodes found in swine. The samples were collected from weaners, fatteners, and sows and examined via flotation (Willis and McMaster), active sedimentation, Ziehl-Neelsen staining as modified by Henricksen, a modified Blagg method, and eggs/oocyst culture. The parasite species detected were *Ascaris suum*, *Trichuris suis*, *Oesophagostomum* spp., *Balantioides coli* (syn. *Balantidium coli*), *Eimeria* spp., and *Cryptosporidium* spp., depending on age category. A dose of 500 mg/kg bw/day of *C. pepo* and 170 mg/kg bw/day of *C. sativum* powders, administered for ten consecutive days, demonstrated a pronounced anthelmintic (pumpkin) and antiprotozoal (coriander) effect against the aforementioned parasites. Future studies are required to ascertain the optimal dose that maximizes their antiparasitic effectiveness. The current study represents the first Romanian report on the in vivo antiparasitic activity of these two plants tested on digestive parasites in swine.

## 1. Introduction

Low-input farming systems can be defined as extensive technological structures that maximize economic and environmental sustainability [1]. In Romania, farmers are raising local breeds (Bazna, Mangalitza, Black of Strei, White of Banat) that are better adapted to processing fibrous feeds, possess a higher tolerance to endemic parasites, have meat with superior organoleptic properties, and are more suitable for organic production [2,3]. Consumers are becoming increasingly interested in buying animal-derived products from free-range farms that are concerned about the wellbeing of their animals [4]. Parasitic infections, for which gastrointestinal protozoa and nematodes are widely considered responsible, are one of the main issues affecting the health and welfare of pigs, decreasing their reproductive performance and productivity [5]. The most important parasites diagnosed in low-input (free-range) farms are *Balantioides coli* (syn. *Balantidium coli*)*, Cryptosporidium* spp., *Eimeria* spp./*Cystoisospora suis*, *Ascaris suum*, *Trichuris suis*, *Strongyloides ransomi,* and *Oesophagostomum* spp. [6,7]. 

Classic antiparasitic drugs (triazine, avermectins, benzimidazole, and imidazothiazoles) are used to treat these parasitic infections in swine [8]. The main drawback of their use is the emergence of antiparasitic resistance to most molecules, along with their residues in animal products [5,9]. The use of traditional herbal medicines is increasing, as medicinal plants have fewer adverse effects than allopathic antiparasitic treatments. Thus, traditional herbal medicine has garnered the attention of numerous researchers, encouraging the screening of certain plants with therapeutic properties in order to assess the effects of their bioactive compounds [10]. Plants generally produce a significant number of secondary metabolites derived from primary ones through biosynthesis, constituting an important resource for several pharmaceutical drugs [11]. Secondary metabolites are divided into three main groups: terpenes (mono- and sesquiterpenes, saponins, and glycosides), phenolic compounds (tannins and flavonoids) and nitrogen-containing compounds (alkaloids and non-protein amino acids), which are believed to represent the main sources of the antiparasitic effect [9]. Phytotherapy is currently viewed as an alternate solution in controlling gastrointestinal parasites in both humans and animals [12]. Phytobiotics are a new class of natural plant-based additives that are highly acceptable among consumers. They can enhance animal productivity along with nutrient absorption, growth performance, and improved digestibility [13].

*Coriandrum sativum* L. is an aromatic and medicinal plant that is widespread throughout the world as a result of being cultivated for its aromatic seeds. It is an annual herb belonging to the Apiaceae family [12,14]. Phytochemical screening of *C. sativum* showed that it contains essential oils, tannins, terpenoids, alkaloids, phenols, flavonoids, fatty acids, sterols, and glycosides. It also contains high levels of proteins, carbohydrates, fibres, and a wide range of minerals and vitamins [15]. Previous pharmacological studies revealed that *C. sativum* plays several biological roles, including antibacterial, antifungal, antiprotozoal, anthelmintic, insecticidal, neuroprotective, antioxidant, cardioprotective, anti-inflammatory, analgesic, antidiabetic, gastroprotective, diuretic, and hepatoprotective effects [10,12,14,15,16,17,18,19]. The observed biological roles can be attributed to the main active compounds in *Coriandrum sativum*, i.e., in its essential oils (linalool, camphor, geranyl acetate, graniol, pinene, and terpine) and oils (petroselinic acid, linoleic acid, and oleic acid) [10,12,14,18,19].

Pumpkins, including *Cucurbita pepo, Cucurbita maxima,* and *Cucurbita moschata,* are gourd squashes belonging to the genus *Cucurbita* and the family Cucurbitaceae. *C. pepo*, the summer squash, is cultivated worldwide as a vegetable [20,21,22]. Pumpkin seeds contain 41.59% oil, 25.4% protein, 5.2% moisture, 25.19% carbohydrates, 5.34% fibre, and 2.49% total ash [21]. Moreover, pumpkin seed oil is used as a nutritional supplement, as it is a natural source of unsaturated fatty acids (omega 9, 6, and 3), lutein, carotenoids, phytosterols, tocopherols, chlorophyll, and trace elements, including selenium and zinc [20,21,23,24,25]. Pumpkin seed extracts are a valuable source of protein and bioactive phytochemicals with positive effects on the general wellbeing, immunity, weight gain, and appetite of chickens [26,27]. Traditionally, this plant is used to treat different medical conditions, including whooping cough, urinary problems, scurvy, rheumatism, haemorrhoids, miscarriage, prostate cancer, constipation, and blindness [21,22,28]. Other medicinal and pharmacological benefits of *C. pepo* seeds include anti-inflammatory, antioxidant, antimicrobial, and antiparasitic effects [22,24,28,29]. These were attributed to the presence of certain classes of compounds, including flavonoids, terpenoids, cardiac glycosides, and cucurbitacin glycosides [22,25,28]. 

The aim of the present study, carried out on two free-range (low-input) Transylvanian farms, was to assess the antiparasitic potential of *C. sativum* and *C. pepo,* present in the Romanian flora, against naturally occurring gastrointestinal parasites in swine.

## 2. Materials and Methods

### 2.1. Biochemical Analyses of Coriandrum sativum and Cucurbita pepo

*C. pepo* (pumpkin) seeds and *C. sativum* (coriander) fruits were used for analysis. Bǎieş et al. [30] described in detail the materials and methods used to analyse the chemical composition of the alcoholic extracts of *C. sativum* fruits and *C. pepo* seeds. The biologically active compounds in these alcoholic plant extracts were analysed by means of high-performance liquid chromatography coupled with mass spectrometry (HPLC-MS). All steps of the procedure were carried out at the Iuliu Haţieganu University of Medicine and Pharmacy in Cluj-Napoca. 

### 2.2. Swine Husbandry

The farms subject to this study were located in Transylvania, a temperate continental climate region positioned in the hills, covered fields, and forests. The samples originated from two low-input swine farms (Farm 1—F1; Farm 2—F2), both raising local Mangalitza and Bazna breeds. In September 2021, when the study was initiated, F1 had a herd of 380 pigs, while F2 had 290 animals. The main water supply was the publicly available drinking water. The barns were cleaned daily throughout the year. Furthermore, before starting the experiment, a rigorous mechanical cleaning was carried out, followed by the disinfection of shelters and paddocks. The outdoor environment was accessible to the pigs at all times. The animals had access to pasture and enrichments (mud bath, straw, roughage, and toys such as chains, bricks, etc.), and the housing area was bordered by an electric fence [6]. 

### 2.3. Experimental Design and Sampling Procedures

Before starting the experiment, a pilot study was conducted on a small number of pigs, through which different doses (according to the literature) of *C. pepo* and *C. sativum* were tested. The feeding behaviour of the animals, antiparasitic efficacy of the plants, and potential side effects were observed.

Each study herd was divided into three age groups: weaners (aged 10 to 11 weeks and weighing 10 to 12 kg), fatteners (aged five to six months and weighing 45 to 50 kg), and sows (aged one to three years and weighing 140 to 150 kg). Each experimental group on which each plant was tested included 10 weaners, 10 fatteners, and 10 sows (*n* = 30 animals), similar to the untreated control groups (*n* = 30 animals) on each farm. In F1, the experiment was performed on Mangalitza pigs, while in F2, Bazna pigs were used. 

*C. pepo* and *C. sativum* were cultivated in Romania and licensed companies provided the plant samples. The seeds of *C. pepo* and fruits of *C. sativum* were ground to a fine powder, resulting in a diet with either pumpkin (1%, 1.35%, or 2.5%) or coriander (0.34%, 0.45%, or 0.85%), along with cereal flour. The feed was provided to the animals according to their age, at a final average concentration calculated by the average body weight of each group (Table 1). The total amount of feed received by each pig was as follows: 0.6 kg, 2 kg, and 3 kg/day per weaner, fattener, and sow, respectively. Thus, pigs belonging to the respective experimental group were administered either 500 mg/kg bw/day of *C. pepo* or 170 mg/kg bw/day of *C. sativum*, divided into two portions, for a period of 10 consecutive days (where bw = body weight). Prior to the beginning of the experiment (day 0), a coproparasitological examination was carried out in order to assess the presence of parasite species. Subsequently, two more coproparasitological examinations were performed, on days 14 and 28 of the study. This procedure was applied on each farm for each plant and each experimental group. 

Faecal samples weighing approximately 20 g each were harvested individually from the rectum of the animals and stored in sterile containers at a temperature of 2–8 °C for 24 to 48 h until testing. The samples were also subjected to macroscopic examination, aiming to detect any visible parasites, before being labelled and stored. Different coproparasitological methods, including flotation (Willis method, McMaster method), centrifugal sedimentation, Ziehl-Neelsen staining as modified by Henricksen, a modified Blagg technique, and faecal cultures (nematode larvae/protozoan oocyst cultures) were involved during testing. The McMaster quantitative method was used to establish the individual intensity of parasitism [6,31,32].

### 2.4. Evaluation of Antiparasitic Efficacy

A faecal egg count reduction test (FECRT) was performed to evaluate the antiparasitic efficacy of *C. sativum* and *C. pepo* using the following formula: FECR (%) = 100 × (1–[T2/T1] × [C1/C2]), with T1 and T2 representing the mean pre- and post-treatment faecal egg counts (FEC) of a treated experimental group, and C1 and C2 representing the mean FEC in the untreated control group before (C1) and after (C2) therapy [33,34]. The same formula was used for both oocysts and cysts.

### 2.5. Ontologies and Ethics Statement 

Table S1 details the ontological analyses of all pathogens, diseases, medicinal plants, and chemical compounds used in the above study, according to the data management plan of the PPILOW (Poultry and Pig Low-input and Organic production systems’ Welfare) project.

The behaviour and clinical condition of the pigs involved were assessed both prior to and throughout the experiment. The bioethical rules for experimentation on animals in both national (law no. 43, 2014) and European (EU Directive 63/2010) legislation were followed.

### 2.6. Statistical Analysis 

The prevalence of each parasite was reported by age group, plant, and farm. We used column graphs to represent the prevalence (Excel ^®^, Microsoft Office 365). A positive test result for *Cryptosporidium* spp. infection was reported as absolute frequency per age group, plant, and farm. The association between a positive result for *Cryptosporidium* spp. and the group (EG vs. CG) was tested with Fisher’s exact test, considering the theoretical frequencies (theoretical frequencies less than 5 in more than 20% of cells).

The distribution of measurements was visually checked with histograms (bell-shaped and symmetrical distribution indicates no deviation from the normal distribution) and box-and-whisker plots (symmetrical box centred around the median, a similar length of the whiskers, and no outliers indicate no deviation from the normal distribution). The graphical method was chosen to investigate the distribution of raw data because the sample size per age group in each farm was small (*n* = 10). According to the distribution, comparisons between cases (experimental group—EG) and controls (CG) by farm and each investigated day (0, 14, and 28) was made with the two-sided Mann–Whitney test at a significance level (α) of 5%. The effectiveness of the investigated plants (day 0, day 14, and day 28) was tested on each group and each farm with the Friedman test, considering a two-sided test and an adjusted α. The maximum possible number of parasites (intensity) per farm and age group (five in our case) was used to adjust the significance level, so the results were considered statistically significant whenever the Friedman test *p*-value was less than 0.01. No post hoc analysis followed the Friedman test due to the limited measurements per investigated day.

The Statistica program (v. 13.5, TIBCO, Tusla, OK, USA) was used to analyse the raw data.

## 3. Results

### 3.1. Analysis of Plant Extracts 

Following the chemical analysis of *C. pepo* and *C. sativum* alcoholic extracts, the bioactive compounds identified were polyphenols (chlorogenic acid, *p*-coumaric acid, ferulic acid, rutoside, syringic acid, and vanillic acid) and sterols (ergosterol, stigmasterol, β-sitosterol, and campesterol) for coriander and tocopherols (γ-tocopherol and Δ-tocopherol) and sterols (stigmasterol, β-sitosterol, and campesterol) for pumpkin. 

### 3.2. Analysis of Antiparasitic Activity of Plants

The coproparasitological examination revealed co-infections of up to five species of gastrointestinal parasites, namely *Balantioides coli, Eimeria* spp., *Cryptosporidium* spp, *Trichuris suis, Ascaris suum*, and *Oesophagostomum* spp. An examination of oocyst/egg cultures revealed that all belonged to the *Eimeria* genus, whereas all L3 larvae (contained within strongylid eggs) belonged to the *Oesophagostomum* genus. Neither centrifugal sedimentation nor the Blagg method gave positive results. The flotation, oocyst/egg culture, and McMaster methods indicated the infection’s prevalence and mean intensity, depending on the age group, farm, and administered plant.

No toxic reactions were recorded in any animal involved in this study. Although this did not represent the objective of the study, clinical observation indicated that animals in both experimental groups consumed their feed better and had a higher growth rate than those in the control groups. This was more obvious in weaners and fatteners.

On both farms and in all three age groups (weaners, fatteners, and sows), identical parasite species and similar co-infection patterns by protozoa and nematodes were found. *Eimeria* spp., *B. coli*, *Cryptosporidium* spp., *A. suum,* and *T. suis* were observed in weaners on both farms. In fatteners from both farms, only *Eimeria* spp., *B. coli*, *A. suum,* and *T. suis* were detected. Lastly, in sows, *Eimeria* spp., *B. coli*, *Cryptosporidium* spp., *Oesophagostomum*, and *A. suum* were identified on both farms.

**Figure 1:**
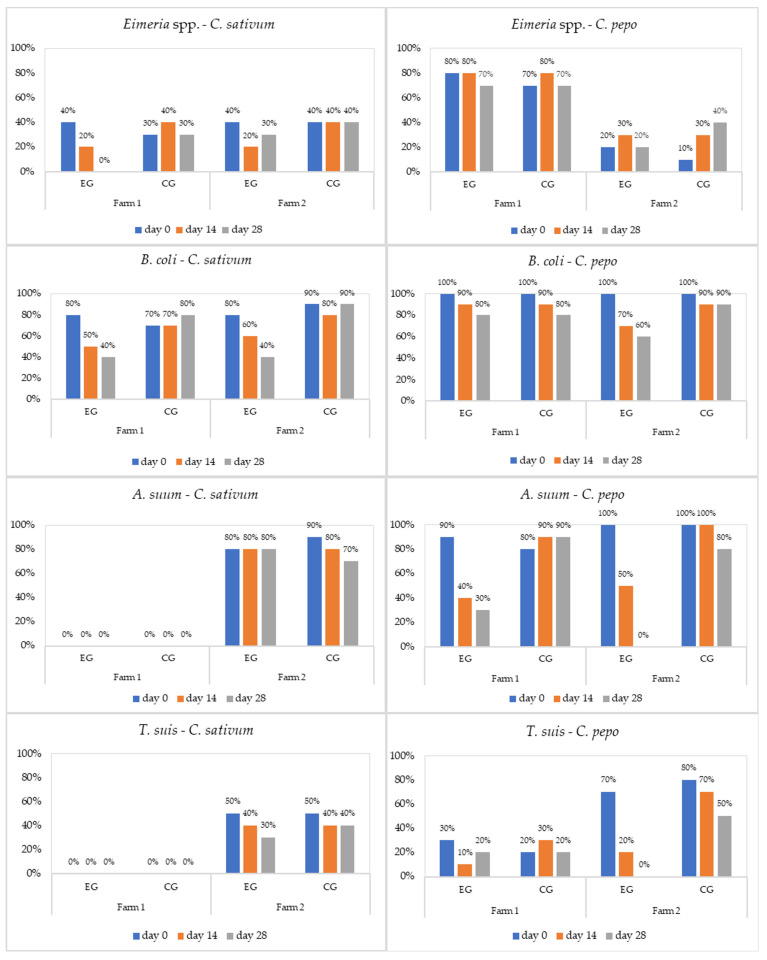
Prevalence (%) of parasites in weaners by treatment (EG = experimental group; CG = control group).

**Figure 2:**
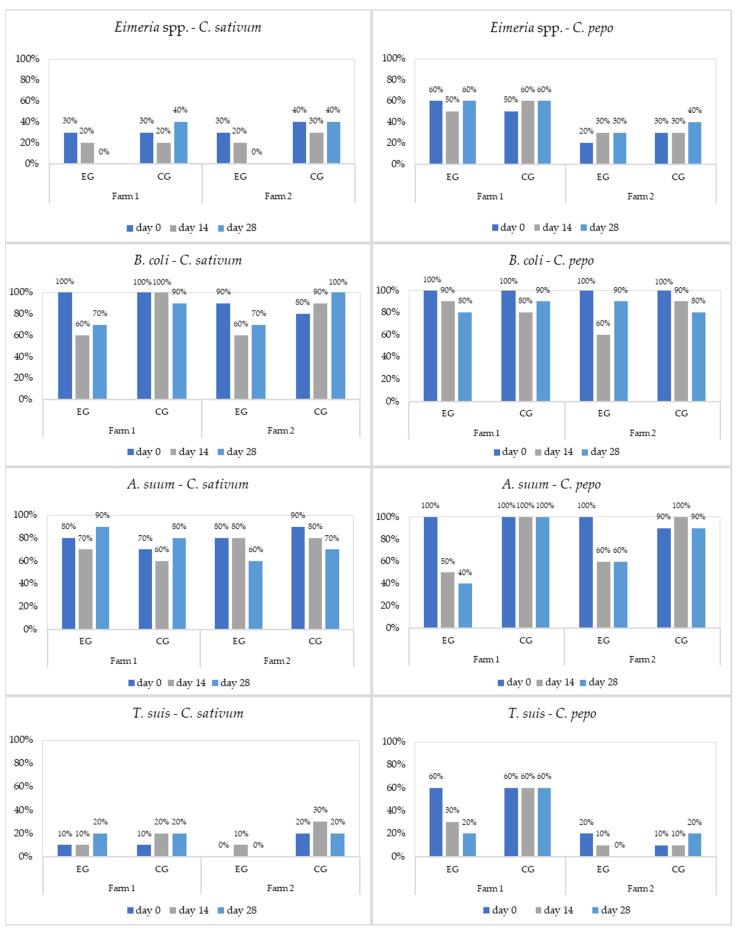
Prevalence (%) of parasites in fatteners by treatment (EG = experimental group; CG = control group).

**Figure 3:**
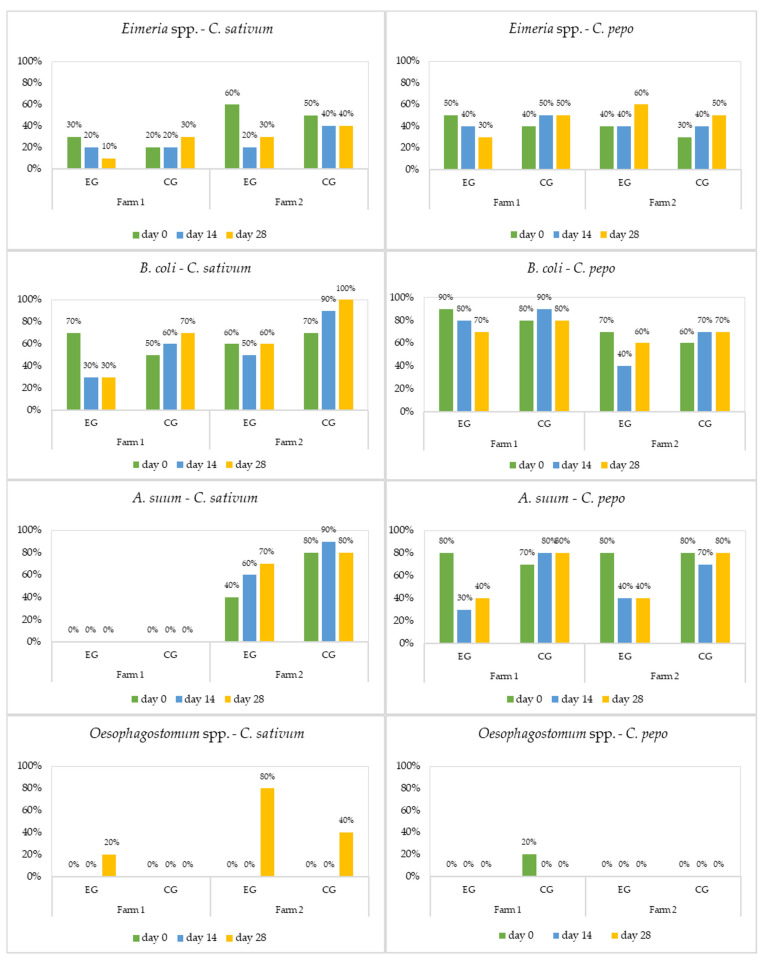
Prevalence (%) of parasites in sows by treatment (EG = experimental group; CG = control group).

*C. pepo* generally demonstrated a strong anthelmintic effect against *A. suum* and *T. suis*, while *C. sativum* had a good antiprotozoal activity against *Eimeria* and *B. coli,* with efficacy according with the breed of pigs and age group (Figure 1, Figure 2 and Figure 3, and Table 2, Table 3, Table 4 and Table 5). 

Statistical significances were found between experimental groups (EGs) and controls (EG 14/ CG 14, EG 28/CG 28) and between EGs (EG 0/EG 14/EG 28) for different plants and farms (pig breeds).

The *C. sativum* group had statistically significant values (SSVs) only for *B. coli*, in both farms and in all age groups (Table 2, Table 3 and Table 4). The *C. pepo* group showed SSVs for *B. coli* in weaners and fatteners from both farms, for *T. suis* in weaners from F2, and for *A. suum* in all age groups from both farms (Table 2, Table 3 and Table 4). 

The studied plants showed limited antiparasitic effects against *Cryptosporidium* spp. and no effects on *Oesophagostomum* spp. Overall, the antiparasitic effects of *C. pepo* and *C. sativum* increased at day 14, with a maximum therapeutic activity at the end of the experiment (day 28).

The therapeutic efficacy (reduction in parasitic intensity, %) of *C. sativum* (CS) and *C. pepo* (CP) against diagnosed parasites in all age groups was as follows: CS = 23.2–79.5%, CP = 2.3–59.5% for *B. coli*; CS = 25.4–100%, CP = 11.6–99.6% for *Eimeria* spp.; CS = 0–0.3%, CP = 50.1–100% for *T. suis;* and CS = 7.2–30.3%, CP = 70.3–100% for *A. suum* (Table 5).

## 4. Discussion

The antiparasitic effects of two aromatic and medicinal plants commonly found in Romania’s flora, namely coriander and pumpkin, were successfully evaluated. We found a higher efficacy of coriander fruits for protozoa and better efficacy of pumpkin seeds for helminths.

The therapeutic potential of medicinal plants renders phytotherapy an alternative to synthetic drugs [11,35]. *C. pepo* and *C. sativum* were found to be effective against several gastrointestinal parasites in swine [11,30,36]. The mechanism of antiparasitic action of *C. sativum* can be attributed to the main bioactive compounds in coriander, i.e., terpenoids, which are components of its essential oils [10,11,12,19], while for pumpkin it was assigned to the presence of cucurbitacins [20,22,25]. Biological compounds such as polyphenols, tocopherols, and sterols present in the studied plants demonstrated effective antiparasitic properties, both in vitro and in vivo [10,11,22,35]. An in-depth analysis of the compounds identified in *C. pepo* and *C. sativum* alcoholic extracts has already been detailed in a previous article [30].

Coriander is a plant with very low toxicity for both humans and animals, even when consumed in large quantities [37]. No information is available on the therapeutic dose of coriander fruits in pigs. We therefore extrapolated the dosage in our experiments based on human reports (100–500 mg/kg/day), along with those for other animal species, namely rats (100–12,000 mg/kg/day) and mice (500–5000 mg/kg/day) [38,39,40,41]. In the present study, a dose of 170 mg/kg/day (divided into two portions) for 10 consecutive days was used. Coriander oil and its major constituent, linalool, have negligible reproductive, neurological, and dermal toxicity in laboratory animals [38,39,40,41]. Limited information exists regarding the safety of orally administered coriander for traditional medicine purposes. Adverse effects associated with the medicinal use of coriander seeds and leaves as empirical treatments have not been documented, although a case report from Iran described endocrine toxicity in a female patient [37,42].

The reported therapeutic dose of *Cucurbita* seeds varies with the animal species, as follows: pigs 5000 mg/kg/day, rats 200–2000 mg/kg/day, mice 300–7600 mg/kg/day, and sheep 5000 mg/kg/day, while in humans it is 100–1000 mg/kg/day [43,44,45,46,47,48,49]. In the current study, a dose of 500 mg/kg/day of pumpkin seeds was administered according to the same therapeutic protocol as that of coriander. *C. pepo* is generally considered safe and non-toxic, and reports of toxicity are scarce [50,51]. Among the only reported side effects—oral allergic syndromes, nausea, diarrhoea, or pruritus in humans and oligospermia and androgen insufficiency in rats—were listed [52,53]. 

*C. sativum* showed statistically significant efficacy for *B. coli* on all age groups regardless of pig breed. *C. pepo* showed efficacy supported by statistical significance on *A. suum* for both pig breeds and age groups, limited efficacy for *B. coli* on any-breed fatteners and Bazna pig weaners, and for *T. suis* on Bazna pig weaners. The absence of significant differences between EG and CG on day 0 for each farm, plant, age category, and diagnosed parasite showed the appropriateness of the study design (Table 2, Table 3 and Table 4).

Eimeriosis is a common parasitic infection in swine that is sporadically related to clinical disease and occasionally linked to diarrhoea and weight loss [54]. Coccidiosis was diagnosed on both farms and in all age groups. *C. sativum* demonstrated a stronger anticoccidial (25.4–100%) effect than *C. pepo* (11.6–96.6%) (Table 5). A commercial herbal formula containing several medicinal plants, including *C. sativum,* effectively controlled experimental coccidiosis in chickens and can potentially be used successfully as a natural anticoccidial drug [55]. Junkuszew et al. [56] established that treating lambs diagnosed with a low intensity of parasitic infection with a mixture of medicinal plants containing *C. pepo* effectively reduced the number of coccidia, also showing a beneficial effect on the growth and body development of the animals. In an in vitro study, *C. pepo* and *C. sativum* alcoholic extracts demonstrated strong anticoccidial effects against *Eimeria* spp. oocysts isolated from swine [57]. Several studies have shown that coriander seed supplements used in poultry feed acted as an alternative to antibiotics and antiparasitics, reducing cholesterol levels and improving both blood parameters and growth performance [58,59]. 

Cryptosporidiosis represents a public health issue. The infection has been reported worldwide as a frequent cause of diarrhoea in both humans and animals [60,61]. In the present study, *Cryptosporidium* spp. was diagnosed in weaners and sows from both farms (Figure 1 and Figure 3). In F2, the plants used were completely inefficacious against these protozoa, while in F1 they showed a weak antiprotozoal efficacy. For *Cryptosporidium,* the results just indicated its presence, whereas the average intensity was not detected, because the quantitative sensitivity of the usual coproparasitological methods when counting oocysts is absent. Both aqueous and alcoholic *C. sativum* extracts had a weak effect on *Cryptosporidium* oocyst shedding, in laboratory infected Balb/c mice in vitro and in vivo [60,61].

*Balantioides coli* (previously known as *Balantidium coli*) is considered the largest protozoan and the only parasitic ciliate known to infect humans. This parasite is often found as a commensal in the lumen of the cecum and large intestine of swine, nonhuman primates, and humans [62]. In one study, the prevalence of the *B. coli* infection on a Danish swine farm increased from 57% in suckling piglets to 100% in most groups of pigs that were several weeks old [63]. In our study, *B. coli* was identified in all age groups, with varying prevalence (30–100%) and intensity. Both plants were efficient against *B. coli*; however, coriander (23.2–79.5%) was superior to pumpkin (2.3–59.5%) (Table 5). The current study serves as a first report on the antiprotozoal effects of *C. pepo* and *C. sativum* against balantidiasis.

*Ascaris suum* is a highly prevalent intestinal nematode in swine worldwide, the infection having a strong negative effect on productivity and health [64]. *A. suum* was only identified in weaners and fatteners from both farms. *C. pepo* (70.3–100%) was very effective against *A. suum*, while *C. sativum* (7.2–30.3%) had a weak anthelmintic effect (Table 5). A previous study established the in vitro anthelmintic effects of *C. sativum* and *C. pepo* ethanolic extracts in different concentrations (0.312–5%) against *A. suum* [30]. *C. sativum* hydroalcoholic extract (4 mg/mL) led to a 45% *A. suum* L2 development inhibition after 20 days of incubation [64]. Pumpkin seed extract used in vitro at a concentration of 54.5% against *A. suum* adults exerted a lethal effect after 11 h 48 min, while at a concentration of 70.5% the time was shortened to 7 h 48 min [65]. *C. pepo* seed oil had a strong anthelmintic efficacy against *Toxocara cati,* inhibiting embryonic development and killing the second-stage larvae, and could be used as an effective alternative for the treatment of toxocarosis [20]. *C. pepo* ethanolic extract demonstrated a proficient anthelmintic effect on embryonating *A. suum* eggs at all tested concentrations (62.5, 125, 250, 500, 1000, and 2000 μg/mL) [66]. In vivo trials showed that pumpkin, used in a dose of 100 mg/chicken, killed 45 ± 2.3 % of *A. galli* worms in the intestinal tract [67]. Aziz et al. [68] demonstrated that treating chickens with a dose of 2000 mg/kg of pumpkin seeds led to a mortality rate in *A. galli* adults similar to that achieved with fenbendazole. Hellawi and Ibrahim [69] showed that pumpkin seed extract had an inhibitory effect on the larval development of *A. galli* and was more effective and safer than levamisole.

*Trichuris suis*, the porcine whipworm, is genetically related to *Trichuris trichiura*, the human whipworm [70]. *T. suis* has shown substantial potential as a treatment for human autoimmune disorders, including inflammatory bowel disease and multiple sclerosis [71]. *T. suis* was diagnosed in weaners and fatteners from F1 and F2. *C. pepo* (50.1–100%) showed a strong anthelmintic effect against this nematode, while *C. sativum* (0–3.3%) had almost no effect (Table 5). No studies on the efficacy of *C. pepo* and *C. sativum* against *T. suis* have been reported before.

*Oesophagostomum* spp., the pig nodular worm, has a worldwide distribution, representing a severe problem for swine health and productivity [72]. *Oesophagostomum* was the last parasite identified. Although this strongyle was diagnosed on both farms, it only infected sows (Figure 3). Because of the infrequent occurrence (low intensity and prevalence) of *Oesophagostomum* spp., it was impossible to come to any reliable statistical interpretation regarding the efficacy of *C. pepo* and *C. sativum* against it (Table 4). Other reports looking at different strongyles outlined the effect of crude aqueous *C. sativum* extract (0.45 and 0.9 g/kg) against *H. contortus* artificial infection in sheep. This extract led to the quantitative reduction of *H. contortus* eggs and adults, comparable to albendazol [73]. Coriander essential oil showed a strong anthelmintic efficacy against ovine gastrointestinal nematodes in vitro by inhibiting egg hatching, larval development, and motility [74,75]. In swine treated with pumpkin seeds at a dose of 5 g/kg repeated three times during one week, the therapeutic efficacy of the plant against *Oesophagostomum* spp. larvae was similar to that of ivermectin [49]. Strickland et al. [76] reported a 65.5% decrease in faecal *H. contortus* egg counts during treatment of sheep with pumpkin seeds, but these increased back to the initial levels as soon as the sheep finished the treatment. Pumpkin seed supplements given at a dose of 5 g/kg/day for 42 consecutive days were not an effective treatment for gastrointestinal nematode infection in kids and lambs [77].

The antiprotozoal effect of the *Cucurbita* genus against numerous parasite species has been documented, including *Entamoeba histolytica*, *Blastocystis* spp., *Dientamoeba fragilis*, *Histomonas meleagridis*, *Tetratrichomonas gallinarum*, *Plasmodium falciparum*, and *Giardia lamblia* [78,79,80,81,82,83]. Furthermore, *C. pepo* extracts showed anthelmintic effects against *Raillietina* spp., *Heterakis* spp., *Heligmosoides bakeri*, *Hymenolepis nana*, *Taenia solium*, and *Aspiculuris tetraptera* [35,84,85,86,87]. Boros et al. [36] showed that *C. sativum* and *C. pepo* alcoholic extracts completely inhibited the mobility of *T. spiralis* and *T. britovi* larvae.

Several studies have shown the antiparasitic effects of *C. sativum*. Coriander essential oil demonstrated significant antiprotozoal effects against *Leishmania amazonensis* and *Leismania infantum* amastigotes and promastigotes, but was not effective against *Leismania chagasi* promastigote [88,89,90]. *C. sativum* presented an anticestodal effect against *Echinococcus granulosus* and *Hymenolepis nana* [12,91]. 

## 5. Conclusions

The present study showed that the use of powdered *C. sativum* fruit and *C. pepo* seed, given at a dose of 170 mg/kg/day and 500 mg/kg/day, respectively, for 10 consecutive days, was efficient against gastrointestinal parasites in swine. Statistical analysis showed that coriander was more effective against protozoa while pumpkin exhibited better efficacy against helminths. Considering all the constraints of Romanian livestock farming, these results are a beacon of hope for better management and welfare practices in low-input swine farms.

The lack of toxicity for *C. pepo* and *C. sativum,* along with our results, allow us to suggest that these medicinal plants could provide the basis for developing a new line of antiparasitic herbal medication, eventually including other plants. Consequently, health and welfare conditions could be greatly improved through the use of these novel therapeutic alternatives. However, additional studies are required to establish the types of bioactive compounds responsible for the antiparasitic properties of these plants and further evaluate the minimum effective dose and the therapeutic protocol tailored to each age category of swine.

In addition, to the best of our knowledge, this is the first ethnopharmacological report on the antiparasitic effects of *C. pepo* and *C. sativum* traditionally used as a novel treatment option in Romania against digestive protozoan and nematode infections in swine. 

## Figures and Tables

**Table 1:** Composition of pig diets by experimental group and age category.

Feed	*C. sativum* Group			*C. pepo* Group		
	Weaners %	Fatteners %	Sows %	Weaners %	Fatteners %	Sows %
corn	38.16	46.05	37.65	37.5	45.15	36
wheat	20	25	25	20	25	25
barley	30	12	20	30	12	20
peas	10	15	15	10	15	15
calcium carbonate	1.5	1.5	1.5	1.5	1.5	1.5
coriander fruits	0.34	0.45	0.85	-	-	-
pumpkin seeds	-	-	-	1	1.35	2.5

**Table 2:** Antiparasitic effects of *C. sativum* and *C. pepo* in weaners.

Parasite	Farm	Group	*p*-Values–Friedman Test					
			*C. sativum*			*C. pepo*		
			Day 0	Day 14	Day 28	Day 0	Day 14	Day 28
*Eimeria* spp.	F1	EG CG	0.0498 ^#^ 0.5818 ^#^			0.7640 ^#^ 0.6387 ^#^		
		*p*-value	0.7337 *	0.3847 *	0.2730 *	0.7723 *	0.3854 *	0.4822 *
	F2	EG CG	0.9170 ^#^ 0.4677 ^#^			0.3679 ^#^ 0.3499 ^#^		
		*p*-value	>0.9999 *	0.3257 *	0.6501 *	0.7337 *	0.7624 *	0.3075 *
*B. coli*	F1	EG CG	0.0076 ^#^ 0.6331 ^#^			0.4724 ^#^ 0.0484 ^#^		
		*p*-value	0.8501 *	0.2123 *	0.0073 *	0.5708 *	0.9699 *	0.7055 *
	F2	EG CG	0.0484 ^#^ 0.4520 ^#^			0.0071 ^#^ 0.2231 ^#^		
		*p*-value	0.9097 ^*^	0.0211 ^*^	0.0013 ^*^	0.4274 ^*^	0.0052 ^*^	0.0233 ^*^
*A. suum*	F1	EG CG	- -			0.0029 ^#^ 0.1561 ^#^		
		*p*-value	-	-	-	0.3847 *	0.0013 *	0.0012 *
	F2	EG CG	0.0125 ^#^ 0.0451 ^#^			0.0001 ^#^ 0.4820 ^#^		
		*p*-value	0.7624 *	0.7913 *	0.2730 *	0.7913 *	0.0002 *	0.0008 *
*T. suis*	F1	EG CG	- -			0.3679 ^#^ 0.7515 ^#^		
		*p*-value*	-	-	-	0.7055 *	0.4057 *	0.9097 *
	F2	EG CG	0.4724 ^#^ 0.9556 ^#^			0.0049 ^#^ 0.0626 ^#^		
		*p*-value	0.9699 *	0.7337 *	0.8798 *	0.6501 *	0.0233 *	0.0640 *
*Cryptosporidium* spp.	F1	EG CG	3 3	3 2	2 1	4 3	2 8	2 8
		*p*-value	0.8142 **	0.6517 **	0.6053 **	0.6749 **	0.7910 **	0.7910 **
	F2	EG CG	1 1	1 1	1 2	1 1	1 2	1 1
		*p*-value	0.7368 **	0.7368 **	0.6053 **	0.7368 **	0.6053 **	0.7368 **

Values represent the *p*-value associated with ^#^ Friedman (three related samples, measurements on day 0, day 14, and day 28) or * Mann–Whitney (two independent samples: EG vs. CG) or ** Fisher’s exact (**) test, except for *Cryptosporidium* spp., where the number of positive tests is displayed within the body of the table; EG = experimental group; CG = control group; “-” = was not diagnosed.

**Table 3:** Antiparasitic effects of *C. sativum* and *C. pepo* in fatteners.

Parasite	Farm	Group	*p*-Values–Friedman Test					
			*C. sativum*			*C. pepo*		
			Day 0	Day 14	Day 28	Day 0	Day 14	Day 28
*Eimeria* spp.	F1	EG CG	0.1738 ^#^ 0.5488 ^#^			0.5308 ^#^ 0.5079 ^#^		
		*p*-value	0.9397 *	0.9699 *	0.1405 *	0.7337 *	0.6231 *	0.6776 *
	F2	EG CG	0.1738 ^#^ 0.6873 ^#^			0.6918 ^#^ 0.7470 ^#^		
		*p*-value	0.7913 *	0.7055 *	0.1405 *	0.7337 *	0.9397 *	0.7913 *
*B. coli*	F1	EG CG	0.0164 ^#^ 0.3469 ^#^			0.0032 ^#^ 0.7165 ^#^		
		*p*-value	>0.9999 *	0.0082 *	0.0257 *	>0.9999 *	0.3447 *	0.0284 *
	F2	EG CG	0.0178 ^#^ 0.8187 ^#^			0.0062 ^#^ 0.9131 ^#^		
		*p*-value	0.5205 *	0.0028 *	0.0041 *	0.9699 *	0.0211 *	0.5205 *
*A. suum*	F1	EG CG	0.0618 ^#^ 0.3796 ^#^			0.0002 ^#^ 0.8975 ^#^		
		*p*-value	0.7624 *	0.7913 *	0.6501 *	0.4057 *	0.0002 *	0.0002 *
	F2	EG CG	0.2545 ^#^ 0.0283 ^#^			0.0003 ^#^ 0.4516 ^#^		
		*p*-value	0.0821 *	0.1306 *	0.1509 *	0.3847 *	0.0002 *	0.0017 *
*T. suis*	F1	Case Control	0.3679 ^#^ 0.3679 ^#^			0.1117 ^#^ 0.7640 ^#^		
		*p*-value	>0.999 *	0.7055	>0.999 *	0.7624 *	0.1212 *	0.0757 *
	F2	EG CG	0.3679 ^#^ n.a.			0.3679 ^#^ n.a.		
		*p*-value *	0.4727 *	0.4497 *	0.4727 *	0.7337 *	>0.9999 *	0.4727 *

Values represent the *p*-value associated with ^#^ Friedman (three related samples, measurements on day 0, day 14, and day 28) or * Mann–Whitney test (two independent samples: EG vs. CG); EG = experimental group; CG = control group; n.a. = not applicable.

**Table 4:** Antiparasitic effects of *C. sativum* and *C. pepo* in sows.

Parasite	Farm	Group	*p*-Values—Friedman Test					
			*C. sativum*			*C. pepo*		
			Day 0	Day 14	Day 28	Day 0	Day 14	Day 28
*Eimeria* spp.	F1	EG CG	0.3679 ^#^ 0.7788 ^#^			0.4668 ^#^ 0.7548 ^#^		
		*p*-value	0.7337 *	0.9699 *	0.4497 *	0.7055 *	0.5454 *	0.3075 *
	F2	EG CG	0.0957 ^#^ 0.8521 ^#^			0.5522 ^#^ 0.3679 ^#^		
		*p*-value	0.4497 *	0.5708 *	0.5967 *	0.6501 *	0.9699 *	0.4497 *
*B. coli*	F1	EG CG	0.0297 ^#^ 0.8789 ^#^			0.1040 ^#^ 0.5811 ^#^		
		*p*-value	0.6232 *	0.1736 *	0.0257 *	0.4963 *	0.9699 *	0.4727 *
	F2	Case Control	0.0344 ^#^ 0.1822 ^#^			0.2609 ^#^ 0.7733 ^#^		
		*p*-value	0.6776 *	0.0015 *	0.0032 *	0.5708 *	0.1736 *	0.6776 *
*Oesophagostomum* spp.	F1	EG CG	- -			0.1353 ^#^ n.a.		
		*p*-value	-	-	0.4727 *	0.4727 *	0.9698 *	0.9698 *
	F2	EG CG	- -			- -		
		*p*-value	-	-	0.0757 ^*^	-	-	-
*A. suum*	F1	EG CG	0.3679 ^#^ 0.3679 ^#^			- -		
		*p*-value	0.7337 *	0.4727 *	0.9699 *	0.3258 *	0.0058 *	0.0073 *
	F2	EG CG	0.0845 ^#^ n.a.			0.0051 ^#^ 0.9726 ^#^		
		*p*-value	0.0073 *	0.0588 *	0.5454 *	0.5454 *	0.0452 *	0.0073 *
*Cryptosporidium* spp.	F1	EG	1	0	0	2	1	0
		CG	1	1	1	1	1	2
	F2	EG	1	1	1	1	1	1
		CG	0	0	1	0	1	0

Values represent the *p*-value associated with ^#^ Friedman (three related samples, measurements on day 0, day 14, and day 28) or * Mann–Whitney test (two independent samples: EG vs. CG), except for *Cryptosporidium* spp., where the number of positive tests is displayed within the body of the table; EG = experimental group; CG = control group; “-” = was not diagnosed; n.a. = not applicable.

**Table 5:** Percentage of faecal egg/oocyst/cyst count reduction (%) recorded on days 14 and 28 post-treatment (using FECR formula).

Parasite	*C. sativum* (Day 14)						*C. sativum* (Day 28)					
	Weaners		Fatteners		Sows		Weaners		Fatteners		Sows	
	F1	F2	F1	F2	F1	F2	F1	F2	F1	F2	F1	F2
*Eimeria* spp.	71.4	72.1	80	30.6	60	41.5	100	25.4	100	100	50	75.7
*B. coli*	29.6	68.9	44.4	62.4	23.2	74.2	84.4	79.5	50.4	20.1	67.4	31.2
*A. suum*	-	18.1	8.1	13.9	-	0	-	30.3	0	7.2	-	0
*T. suis*	-	0	0	0	-	-	-	3.3	0	0	-	-
**Parasite**	***C. pepo* (day 14)**						***C. pepo* (day 28)**					
	**Weaners**		**Fatteners**		**Sows**		**Weaners**		**Fatteners**		**Sows**	
	**F1**	**F2**	**F1**	**F2**	**F1**	**F2**	**F1**	**F2**	**F1**	**F2**	**F1**	**F2**
*Eimeria* spp.	11.6	96.6	13.9	33.3	45.4	0	24.9	94.7	35.9	0	61.1	0
*B. coli*	2.3	59.5	22.9	54.9	3.0	30.1	0	34.1	45.1	24.8	33.6	22.2
*A. suum*	77.4	80.9	83.5	79.7	87.1	70.3	79.7	100	84.5	95.9	85.9	88.9
*T. suis*	91.6	80.7	50.1	75.0	-	-	91.0	100	57.7	100	-	-

F1 = Farm 1; F2 = Farm 2; “-” = was not diagnosed; “0” = was identified, but had no efficacy.

## Data Availability

Not applicable.

## Supplementary Materials

The following supporting information can be downloaded at: https://www.mdpi.com/article/10.3390/microorganisms11051230/s1, Table S1. Ontologies/pathogens, diseases, medicinal plants, and chemical compounds used in experiment.
