# Supplementary material for: The Effects of Coriandrum sativum L. and Cucurbita pepo L. against Gastrointestinal Parasites in Swine: An In Vivo Study
Source: Microorganisms. 2023 May 6;11(5):1230. doi: 10.3390/microorganisms11051230 (PMC10223014; doi:10.3390/microorganisms11051230)
Supplement: Supplementary file 1 [file microorganisms-11-01230-s001.zip › microorganisms-2303879-supplementary.pdf]

Supplementary Data

Table S1. Ontologies/pathogens, diseases, medicinal plants and chemical compounds used in experiment.

Traits	ATOL*, AHOL**, OPL***, IPNI****, and ChEBI***** References	
**Parasite load traits**	Parasite Oocysts (OPG)	Oocyst Stage
	Parasite Cysts	Cyst Stage
	Parasite Eggs (EPG)	Egg Stage
**Parasite used**	*Eimeria* spp.	AHOL_0004070
	Balantioides coli	AHOL_0004016
	Ascaris suum	AHOL_0004179
	Trichuris suis	AHOL_0004186
	Oesophagostomum spp.	AHOL_0004181
	Cryptosporidium spp.	AHOL_0004175
**Disease description**	Ascaridiosis	AHOL_0005382
	Coccidiosis	AHOL_0005374
	Cryptosporidiosis	AHOL_0005377
**Medicinal plants used**	*Coriandrum sativum* L.	840760-1
	Cucurbita pepo L.	292416-1
**Chemical compounds detected**	Polyphenols (μg/mL)	26195
	Tocopherols (ng/mL	135821
	Sterols (μg/mL)	15889

*Traits in reference to the ontology ATOL: https://www.atol-o.com/en/atol-2/; **Traits in reference to the ontology AHOL: https://www.atol-onntologytology.com/ahol/; *** Ontology for Parasite Life cycle: http://wiki.aiisc.ai/index.php/Ontology_for_Parasite_Life_Cycle; **** International Plant Names Index-IPNI : *https://www.ipni.org/p/3;* ***** Chemical Entities of Biological Interest-ChEBI: https://www.ebi.ac.uk/chebi/.
